# Supplementary material for: End Sequence Analysis Toolkit (ESAT) expands the extractable information from single-cell RNA-seq data
Source: Genome Res. 2016 Oct;26(10):1397–410. doi: 10.1101/gr.207902.116 (PMC5052061; doi:10.1101/gr.207902.116)
Supplement: Supplemental Material [file supp_gr.207902.116_Supp_Materials.tar.gz › ESAT-esat_v0.1/doc/html/classumms_1_1core_1_1model_1_1score_1_1_end_in_window_count_score.html]

ESAT: umms.core.model.score.EndInWindowCountScore Class Reference


ESAT


- Main Page
- Related Pages
- Packages
- Classes
- Files

- Class List
- Class Index
- Class Hierarchy
- Class Members

All Classes Namespaces Files Functions Variables Enumerator Pages

- umms
- core
- model
- score
- EndInWindowCountScore

Public Member Functions |
List of all members

umms.core.model.score.EndInWindowCountScore Class Reference

Inheritance diagram for umms.core.model.score.EndInWindowCountScore:

[legend]

Collaboration diagram for umms.core.model.score.EndInWindowCountScore:

[legend]

|  |  |
| --- | --- |
| Public Member Functions | |
|  | EndInWindowCountScore (Annotation t) |
|  | |
|  | EndInWindowCountScore (AlignmentModel model, Annotation annotation) |
|  | |
| double | getScore () |
|  | |
| double | getCount () |
|  | |
| int | compareTo (EndInWindowCountScore o) |
|  | |
| void | setCount (double count) |
|  | |
| Public Member Functions inherited from umms.core.model.score.WindowScore.AbstractWindowScore | |
|  | AbstractWindowScore (Annotation t) |
|  | |
| Annotation | getAnnotation () |
|  | |

|  |  |
| --- | --- |
| Additional Inherited Members | |
| Protected Attributes inherited from umms.core.model.score.WindowScore.AbstractWindowScore | |
| Annotation | annotation |
|  | |

## Constructor & Destructor Documentation

|  |  |  |  |  |  |
| --- | --- | --- | --- | --- | --- |
| umms.core.model.score.EndInWindowCountScore.EndInWindowCountScore | ( | Annotation | *t* | ) |  |

|  |  |  |  |
| --- | --- | --- | --- |
| umms.core.model.score.EndInWindowCountScore.EndInWindowCountScore | ( | AlignmentModel | *model*, |
|  |  | Annotation | *annotation* |
|  | ) |  |  |

Here is the call graph for this function:

## Member Function Documentation

|  |  |  |  |  |  |
| --- | --- | --- | --- | --- | --- |
| int umms.core.model.score.EndInWindowCountScore.compareTo | ( | EndInWindowCountScore | *o* | ) |  |

Here is the call graph for this function:

|  |  |  |  |  |
| --- | --- | --- | --- | --- |
| double umms.core.model.score.EndInWindowCountScore.getCount | ( |  | ) |  |

Here is the caller graph for this function:

|  |  |  |  |  |
| --- | --- | --- | --- | --- |
| double umms.core.model.score.EndInWindowCountScore.getScore | ( |  | ) |  |

Returns the number of reads overlapping this region

Returns
:   number of reds overlapping this window Returns the "score" as defined by each subclass (e.g. count, ratio, sum of annotation scores, etc.)

Implements umms.core.model.score.WindowScore.

Here is the call graph for this function:

|  |  |  |  |  |  |
| --- | --- | --- | --- | --- | --- |
| void umms.core.model.score.EndInWindowCountScore.setCount | ( | double | *count* | ) |  |

Here is the caller graph for this function:

---

The documentation for this class was generated from the following file:

- java/umms/core/model/score/EndInWindowCountScore.java


---

Generated on Fri Aug 15 2014 15:11:12 for ESAT by  

 1.8.7
